# Total intravenous anaesthesia with remimazolam in a patient with progressive supranuclear palsy

**DOI:** 10.1002/anr3.70011

**Published:** 2025-05-07

**Authors:** T. Oshida, T. Taniguchi

**Affiliations:** ^1^ Department of Anaesthesia and Palliative Care Kanazawa University Hospital Kanazawa Japan

**Keywords:** general anaesthesia, progressive supranuclear palsy, remimazolam

## Abstract

Progressive supranuclear palsy is a neurodegenerative disease of unknown aetiology; few reports address its anaesthetic management. Remimazolam, a recently approved short‐acting benzodiazepine, was used in combination with remifentanil for total intravenous anaesthesia during open cholecystectomy in a patient with progressive supranuclear palsy. Due to its cardiovascular stable profile and rapid reversibility with flumazenil, remimazolam may serve as a viable option for general anaesthesia in patients with this condition.

## Introduction

Both general and neuraxial anaesthesia are viable options for patients with progressive supranuclear palsy (PSP). This case report describes a patient undergoing open cholecystectomy for which total intravenous anaesthesia with remimazolam, selected for its potential to provide rapid emergence with the use of flumazenil, was administered. The peri‐operative period was uneventful.

## Report

The patient was a 76‐year‐old man with a history of appendicectomy as a teenager and abdominal surgery following a traffic accident in his forties. He had developed dysuria and gait disturbance and was diagnosed with PSP three years previously. Although levodopa/carbidopa treatment was initiated, his condition progressively worsened. The patient was able to communicate, but his Barthel Index score was only 10 points, and he required assistance in almost all daily activities. The Barthel Index is a widely used tool for assessing a person's ability to perform basic activities of daily living. The total score ranges from 0 (complete dependence) to 100 (complete independence).

The patient was diagnosed with cholecystitis following fever and impaired consciousness, but surgery was deemed high risk. Percutaneous transhepatic gallbladder drainage was performed and antimicrobial agents initiated. At this time, deep vein thromboses of both lower limbs were diagnosed for which apixaban was started. His condition gradually improved, but as the drainage output decreased over time, an open cholecystectomy was scheduled one month after the onset of cholecystitis. As the operation was an open surgery, a combination of general anaesthesia and epidural anaesthesia was initially considered. Japanese regional anaesthesia guidelines require a 72‐h withdrawal of apixaban when epidural anaesthesia is performed [1]. However, the apixaban withdrawal period for this patient was only 48 h. As postoperative pain is manageable with abdominal wall nerve block and continuous intravenous fentanyl infusion as an alternative to epidural anaesthesia, we decided to proceed with surgery as planned. Therefore, the anaesthetic plan was changed to general anaesthesia and nerve block of the abdominal wall. Remimazolam was chosen due to minimal cardiovascular effects and its rapid reversal with flumazenil.

Invasive arterial pressure monitoring was initiated before induction of anaesthesia. Anaesthesia was induced with remimazolam at a rate of 2.5 mg.kg^−1^.h^−1^ and remifentanil at a rate of 0.13 μg.kg^−1^.h^−1^. Two minutes later, as the patient lost consciousness, 60 mg rocuronium was administered. Mask ventilation and tracheal intubation using videolaryngoscopy were performed without difficulty. The remimazolam infusion rate was reduced to 0.4 mg.kg^−1^.h^−1^ after the onset of general anaesthesia and subsequently adjusted to maintain a bispectral index value of 40–60. The remifentanil infusion rate was adjusted at a rate of 0.08–0.24 μg.kg^−1^.h^−1^. The patient remained cardiovascularly stable without the need to use vasoactive support. The train‐of‐four (TOF) ratio recovered to 90% by 2 h after the initial dose of rocuronium, when a further 10 mg was administered. At the time of peritoneal closure, the patient had a TOF ratio of 60%, but closure was achieved without difficulty. The operation took longer than usual due to a significant amount of intra‐abdominal adhesions. Nevertheless, total blood loss was 80 ml. The postoperative analgesia plan included rectus sheath and transversus abdominis blocks alongside intravenous paracetamol and continuous fentanyl infusion. Five minutes after discontinuing remimazolam and remifentanil, the patient was breathing spontaneously and was opening his eyes to verbal request. After waiting for a few minutes, he did not exhibit any other responses to verbal stimuli, which led us to believe he was not sufficiently conscious. We decided to administer flumazenil 0.5 mg, and within a minute, the patient was able to open his eyes spontaneously and follow further verbal commands. As the TOF ratio exceeded 90%, he was extubated without rocuronium reversal. Following tracheal extubation, he was transferred to the general ward without any significant complications.

## Discussion

Progressive supranuclear palsy is a neurodegenerative disease characterised by vertical supranuclear eye movement disorder, somatic muscle rigidity and cognitive impairment; with a prevalence of 5.82 per 100,000 people in Japan [2]. The aetiology remains unknown, with an average disease duration of 5–9 years. The most common cause of death in patients with PSP is pneumonia, which is associated with dysphagia [2]. Although no treatment has been established to slow disease progression, levodopa/carbidopa has been employed to alleviate motor symptoms [2].

Currently, no established guidelines exist regarding the anaesthetic management of patients with PSP. Various anaesthetic techniques, including general anaesthesia, epidural anaesthesia, subarachnoid anaesthesia and peripheral nerve blocks have been reported [3, 4]. In this case, due to inadequate pre‐operative withdrawal of apixaban, general anaesthesia combined with abdominal wall blocks was chosen. Patients with PSP may present airway management challenges due to stridor or cervical deformity during general anaesthesia [5]; thus, careful attention is required during induction. Additionally, as dysphagia is common in patients with PSP, poor postoperative emergence increases the risk of sputum retention and aspiration pneumonia [3]. Inhalational anaesthetic agents can be administered to PSP patients. However, there have been reports of patients who could not be extubated in the operating theatre due to poor emergence after general anaesthesia with sevoflurane, requiring admission to the intensive care unit [4]. Patients with PSP often take levodopa/carbidopa, and discontinuation of these drugs during the peri‐operative period may lead to intractable hypotension resulting from reduced endogenous catecholamine levels [6]. Therefore, careful monitoring for hypotension is essential during the peri‐operative period. Although propofol can be used in PSP patients, remimazolam offers the additional benefits of reversibility with flumazenil and a better cardiovascular profile than propofol [7]. While no previous reports describe the use of remimazolam in patients with PSP, benzodiazepines, such as diazepam and clonazepam, are sometimes used to treat dystonia and myoclonus, supporting the use of remimazolam in this context [8]. However, excessive salivation has been associated with remimazolam use, and caution is warranted, as this can increase the risk of laryngospasm and aspiration [9].

Rocuronium can be safely used in patients with PSP [4]. Intra‐operative monitoring of the neuromuscular block was used and recovery was normal. We did not administer a reversal agent, as the patient had a TOF ratio exceeding 90% at the time of tracheal extubation. It is important to note that patients with PSP are at high risk of aspiration and choking postoperatively due to their impaired swallowing function.

We selected remimazolam for general anaesthesia in a patient with PSP and managed the peri‐operative period safely without significant complications. Remimazolam should be considered a viable option for general anaesthesia for patients with PSP as it provides rapid emergence when reversed with flumazenil and has minimal cardiovascular effects.
